# Near-Infrared Light Increases Functional Connectivity with a Non-thermal Mechanism

**DOI:** 10.1093/texcom/tgaa004

**Published:** 2020-03-19

**Authors:** Grzegorz M Dmochowski, Ahmed Duke Shereen, Destiny Berisha, Jacek P Dmochowski

**Affiliations:** 1 Princess Margaret Cancer Centre, Toronto, ON M5G 1L7, Canada; 2 Advanced Science Research Center, Graduate Center of the City University of New York, New York, NY 10031, USA; 3 Department of Biomedical Engineering, City College of New York, New York, NY 10031, USA

**Keywords:** fMRI, functional connectivity, low-level laser therapy, neuromodulation, photobiomodulation

## Abstract

Although techniques for noninvasive brain stimulation are under intense investigation, an approach that has received limited attention is transcranial photobiomodulation (tPBM), the delivery of near-infrared light to the brain with a laser or light-emitting diode directed at the scalp. Here we employed functional magnetic resonance imaging to measure the blood-oxygenation-level–dependent signal in *n* = 20 healthy human participants while concurrently stimulating their right frontal pole with a near-infrared laser. Functional connectivity with the illuminated region increased by up to 15% during stimulation, with a quarter of all connections experiencing a significant increase. The time course of connectivity exhibited a sharp rise approximately 1 min after illumination onset. Brain-wide connectivity increases were also observed, with connections involving the stimulated hemisphere showing a significantly larger increase than those in the contralateral hemisphere. We subsequently employed magnetic resonance thermometry to measure brain temperature during tPBM (separate cohort, *n* = 20) and found no significant temperature differences between active and sham stimulation. Our findings suggest that near-infrared light synchronizes brain activity with a nonthermal mechanism, underscoring the promise of tPBM as a new technique for stimulating brain function.

## Introduction

Causal interventions of neural activity are necessary to identify the brain circuits underlying complex behaviors and also to spur the development of nonsystemic therapies for neurological and psychiatric disorders. The most popular approaches employ mild electric currents (Nitsche et al. 2008), magnetic fields (Hallett 2007), or ultrasonic pressure waves (Naor et al. 2016). These techniques are aimed at directly evoking neuronal activation or modulating cortical excitability. In contrast, a more recent neuromodulation technique employs light to target the brain’s energy metabolism pathway. Termed transcranial photobiomodulation (tPBM), this relatively unknown approach delivers near-infrared light to the brain via transcranial transmission with a laser or light-emitting diode (LED) (Hamblin 2016). The purported mechanism of action in tPBM is the absorption of light by cytochrome c oxidase (CCO), the terminal enzyme in the mitochondrial electron transport chain, leading to increased energy metabolism (Cassano et al. 2016; Salehpour et al. 2018b). Findings from small animal models suggest that tPBM increases cerebral blood flow (CBF) (Uozumi et al. 2010) and cortical ATP (Mochizuki-Oda et al. 2002), while reducing inflammatory markers (Moreira et al. 2009; Zhang et al. 2014) and apoptosis (Wong-Riley et al. 2005; Yu et al. 2015; Salehpour et al. 2017). tPBM has also been reported to ameliorate amyloid beta accompanying neurodegeneration (Lu et al. 2017) and oxidative damage from sleep deprivation (Salehpour et al. 2018a). It is largely unknown whether such effects could be achieved in human.

It is important to determine whether tPBM represents a viable form of noninvasive brain stimulation, and if so, through what mechanism of action. To date, there are limited reports of the neurophysiological effects of tPBM in the human brain. Tian et al. (2016) employed near-infrared resonance spectroscopy (NIRS) and reported increased cerebral oxygenation in both hemispheres during tPBM. A recent report suggests that tPBM increases the power of electrophysiological oscillations as measured by the scalp electroencephalogram (EEG) (Wang et al. 2019). Behavioral investigations have reported that tPBM may improve performance on cognitive tests of working memory and attention (Barrett and Gonzalez-Lima 2013; Hwang et al. 2016; Blanco et al. 2017a, 2017b). Nevertheless, the ability of tPBM to accelerate cerebral energy metabolism in humans remains largely untested: does the human brain respond metabolically to tPBM, and if so, with what temporal dynamic? Moreover, given that tPBM involves depositing energy into the brain, it is critical to ascertain whether heating is involved in any observed effects.

Functional magnetic resonance imaging (fMRI) affords the opportunity to test the merit of tPBM in the human brain. In particular, the blood-oxygenation-level–dependent (BOLD) signal (Ogawa et al. 1990) is closely linked to energy metabolism, with both CBF and the cerebral metabolic rate of oxygen (CMRO_2_) contributing to the measured signal (Buxton 2013). BOLD has traditionally been employed as an indirect measure of neural activation via neurovascular coupling. At rest, a stereotyped pattern of temporal correlations among the BOLD signals of distributed brain regions is observed, in what is known as resting-state functional connectivity (FC) (Fox and Raichle 2007). In addition to BOLD imaging, magnetic resonance (MR) thermometry (Rieke and Butts Pauly 2008) permits the measurement of brain tissue temperature during stimulation, such that the presence and magnitude of heating can be determined.

Three recent reports have employed fMRI to shed light on the aftereffects of tPBM. Chao (2019) applied tPBM home treatments to a small cohort of dementia patients, finding increased FC between the posterior cingulate and lateral parietal cortex at the conclusion of the 12-week study. Increased FC within the default-mode network was found after tPBM in a small cohort of chronic stroke patients (Naeser et al. 2019). In a study of tPBM in healthy participants, El Khoury et al. (2019) report reductions in somatosensory evoked activity and increased FC within the parietal association cortex. However, the acute effects of tPBM on human brain activity have yet to be probed withfMRI.

Here we conducted the first fMRI measurements during tPBM in humans. We recruited *n =* 20 healthy participants to receive a 10-min application of tPBM to the right frontal pole while recording their hemodynamic activity with BOLD-fMRI. We found a robust effect on FC with the illuminated region: a quarter of all connections were significantly increased, with the increase as high as 15%. The time course of FC exhibited a sharp increase shortly after illumination onset. We also found enhanced FC between regions outside of the illuminated area, with a significantly larger increase in the stimulated hemisphere. We subsequently employed MR thermometry to measure brain temperature changes during tPBM with the same parameters as the BOLD study (separate cohort of *n* = 20). We failed to detect a thermal effect of tPBM, with the temperature in the illuminated region not varying significantly from sham stimulation at any time points.

## Materials and Methods

### Subjects

All experimental procedures were approved by the Institutional Review Board of the City University of New York. We recruited *n* = 40 participants (20 females) from the local New York City population. In an attempt to achieve uniform baseline measures of CBF and CMRO_2_ in our sample, only subjects aged 18–40 were considered: the CMRO_2_ has been found to decrease monotonically with age (Yamaguchi et al. 1986; Marchal et al. 1992). The mean age of the participants was 24.8 *±* 4.6 years. During recruitment, we employed those exclusionary criteria common to MRI (e.g., subjects with cardiac pacemakers, neurostimulation systems, or claustrophobia were excluded). All subjects completed the experiments and there were no major adverse effects. In the BOLD-fMRI study, one subject complained of a headache following the scan, which may have been caused by the headgear that was worn throughout the (*∼*1 h) experiment.

### Experimental Design

#### BOLD-fMRI Study

A total of *n* = 20 subjects (10 females) participated. The BOLD signal was continuously acquired for 30 min. The laser was turned on 10 min after the onset of the scan and remained active for a 10-min duration. Subjects were not made aware of when the laser was turned on or for how long. From verbal postexperimental surveys, subjects did not perceive any sensations (thermal or otherwise) during illumination.

#### MR Thermometry Study

A total of *n* = 20 subjects (10 females) participated. All subjects performed 2 sessions in succession. Throughout each 20.5-min session, brain temperature was measured with a temperature-sensitive MRI sequence. In one session (“active”), the laser was turned on 172 s (21 TRs) into the scan and remained on for 10 min. In the other session (“sham”), the laser remained off throughout. As the switch controlling the laser was housed in the MRI control room, subjects could not see or hear the operation of the device. The order of sham and active sessions was randomized and counterbalanced across subjects.

### tPBM

tPBM was applied at a wavelength of 808 nm, selected based on a previous study that demonstrated an improvement in cognitive task performance after tPBM in healthy volunteers (Barrett and Gonzalez-Lima 2013). This wavelength falls within the so-called “optical window” in which absorption by common chromophores (water, melanin, and hemoglobin) is low (Hamblin and Demidova 2006), allowing for deeper penetration through the human scalp and skull and into the brain as compared with other candidate wavelengths (Tedford et al. 2015; Pitzschke et al. 2015). A class IV 10-W diode laser (Ultralasers MDL-N-808-10 000) powered by a laser driver (Ultralasers PSU-H-LED) provided the monochromatic light. A calibrated photodetector was employed to set the laser power to 250 mW prior to each experiment. Given a 1-cm diameter aperture in the headgear worn by participants, this resulted in an intensity of 318 mW/cm^2^, which is within the ANSI (American National Standards Institute) safety limit for human tissue (330 mW/cm^2^). As the duration of stimulation was set to 10 min, the total incident light energy was 150 J. It should be noted that the coherent nature of the laser is not believed to contribute to the action of tPBM. A given photon undergoes multiple scattering events on its path to the brain, thereby losing its original phase information. LEDs have elicited effects in previous PBM experiments (Chung et al. 2012). A laser was employed in this case to facilitate light delivery at the desired power.

The laser output was coupled directly into a custom made, multimode optical fiber (Thorlabs FT400EMT) whose core diameter was 400 μm. To ensure MR compatibility, the distal end of the optical fiber was fitted with a ceramic ferrule that was affixed to a custom 3D-printed headgear worn by participants. The headgear, measuring 5.5 cm *×* 3.2 cm *×* 2.7 cm, contained a clamp, which secured the ferrule. The headgear was secured against the subject’s head such that its aperture was flush against the forehead. The aperture was centered at location “Fp2” (right frontal pole) of the 10/20 standard system for EEG (Jasper 1958), where each participant’s Fp2 location was measured and marked on the scalp prior to the experiment. Note that stimulating the forehead yields better penetration through the scalp due to the absence of scattering by the hair. For all but 2 subjects, vitamin E markers were placed on the headgear so that the location of light incidence could be registered with the anatomical MRI. All subjects wore protective goggles in addition to the laser headgear throughout all experiments.

### Estimating the Illuminated Region

The primary region-of-interest (ROI), referred to herein as the “illuminated region,” was constructed based on numerical simulations of light propagation through the human head. We employed the Monte Carlo Multi-Layered (MCML) software (Wang et al. 1995), which models an infinitely narrow photon beam normally incident on multiple layers of turbid material. The 4 layers here corresponded to scalp, skull, cerebrospinal fluid (CSF), and brain. The properties of the model are listed in Supplementary Table S1. The output of the simulation was the light absorption (J/cm^3^) in the volume, computed over a discrete grid in cylindrical coordinate space. To define the illuminated region, we computed the smallest cylinder that contained at least 99% of the total absorption. An exhaustive search procedure produced a cylindrical ROI with a radial extent of 2.1 cm and axial extent of 3.9 cm. This ROI was “projected” onto each subject’s anatomical MRI from the position of the aperture.

### MRI, fMRI, and MR Thermometry

#### BOLD-fMRI

Imaging was performed with a Siemens Magnetom Skyra 3 Tesla scanner. A 16-channel transmit/receive head coil was used for data acquisition. Structural images were acquired with a T1-weighted MPRAGE sequence (field-of-view (FOV) 230 mm, in plane resolution 256 × 256, 224 slices with a thickness of 0.9 mm, TI = 1000 ms). Functional BOLD scans were acquired with a multi-echo echo-planar imaging (EPI) sequence (FOV 228 mm, in plane resolution 90 × 90, 60 slices with a thickness of 2.5 mm, TR = 2800 ms, flip angle = 82 degrees). The 3 echo times were 12.8 ms, 34.3 ms, and 55.6 ms, which allowed for the characterization of the T2^*^ decay of the BOLD signal (Posse et al. 1999). The duration of the BOLD scans was 30 min (645 volumes). Subjects were instructed to rest but stay awake and to not think about anything in particular.

#### MR Thermometry

Imaging was performed with a Siemens Prisma 3 Tesla scanner. Signal excitation was performed with the built-in body coil, and a 20-channel phased array head/neck coil was used for data acquisition. Structural images were acquired with a T1-weighted MPRAGE sequence (FOV 256 mm in read and 240 mm in phase-encode directions, in-plane matrix size of 256 × 240, 208 sagittal slices with a thickness of 1 mm, TR/TE/TI = 2400/2.15/1000 ms, flip angle = 8 degrees, ×2 GRAPPA acceleration factor, fat suppression using fast water excitation, and a total acquisition time of 5:26 min). To measure brain temperature changes during tPBM, we employed a custom 3D EPI phase-difference imaging sequence that exploits the temperature dependence of the proton resonance frequency (PRF) (Ishihara et al. 1995; Rieke and Butts Pauly 2008). Baseline phase was first measured as an average over 3 initial frames, and subsequent image phases were referenced to this baseline phase. The phase difference has a linear dependence on the temperature change from baseline (Rieke and Butts Pauly 2008). Thermometry scans employed an FOV of 192 mm in both read and phase directions, an in-plane matrix size of 64 × 64, with 32 interleaved slices per slab of thickness of 3 mm, TR/TE = 25/17 ms, flip angle = 10 degrees, EPI factor = 7, echo spacing = 0.93 ms, bandwidth = 1302 Hz/Px, with no fat suppression or accelerated imaging used. The duration of the scans was 20.5 min (150 volumes).

### BOLD Preprocessing

Preprocessing of BOLD data was performed with the AfNI software package (Version 17.3.03) (Cox 1996), scripted in the Matlab programming language (Math-works). The anatomical image was first skull stripped using the 3dSkullStrip function, whose output was then used to create a brain mask via the 3dAutomask function. The anatomical image was segmented to produce tissue masks for the CSF, gray matter, and white matter via the 3dSeg routine. The first 3 frames of all BOLD series were excluded from analysis. The function 3dDespike was applied to the raw BOLD series to remove large transients. Slices were aligned to the onset of each TR. Motion correction was performed by aligning each volume of the BOLD series to a reference volume (i.e., frame 3). We registered the BOLD data to the corresponding anatomical image and applied a nonlinear warping procedure to then transform the data to the Talairach coordinate space. Spatial smoothing with a full-width-half-max (FWHM) of 4 mm was then performed. Each voxel’s time series was normalized to a mean of 100. The time series of the motion alignment parameters and their derivatives were linearly regressed out of the data. The BOLD was then band-pass filtered to the range 0.01–0.1 Hz. Volumes during which the derivative of the motion parameters exceeded a norm of 0.3 were censored. Volumes during which >15% of the voxels were identified as outliers, defined as those samples exceeding 5.8 times the median absolute deviation, were also censored.

### FC Analysis

FC was performed on time series from 151 ROIs formed by the union of the Destrieux atlas (75 ROIs in each hemisphere) (Destrieux et al. 2010) and the illuminated region. The time series of gray matter voxels comprising each ROI were averaged prior to connectivity analysis. As we observed a transient increase in FC during the first 5 min of recording, we retained only the second half of the pre-illumination period for analysis (Supplementary Fig. S1). In other words, preillumination values were measured once the data reached a stable level. For each of the 3 experimental time segments: before illumination (5 min), during illumination (10 min), and immediately following illumination (10 min), we measured FC as the Pearson correlation coefficient between each unique pair of ROI time courses. Correlation coefficients were Fisher transformed prior to all statistical tests. We also measured the total connectivity of the illuminated region as the mean correlation between the illuminated region and each of its 150 connecting ROIs (mean across 150 connecting ROIs).

We tested for significant effects of tPBM on FC with the permutation testing approach advocated in the field of dynamic FC (dFC) (Preti et al. 2017; Handwerker et al. 2012). Surrogate data records were constructed by permuting the phase spectrum of voxel time series, in all cases preserving spatial correlations among voxels and autocorrelations within time series. From this, we were able to construct null distributions of the group-level difference in FC between the illumination and preillumination period, and also between the postillumination and preillumination period. We also employed the permutation test approach when testing for changes in total connectivity and brain-wide connectivity. In other words, we performed the same sequence of operations as was carried out on the actual data but on the permuted data. In all cases, we corrected for multiple comparisons by controlling the false discovery rate (FDR) at 0.05. When testing for the presence of significantly larger effects in the right hemisphere, we conducted a 2-way repeated-measures analysis of variance (ANOVA) with hemisphere (left hemisphere–left hemisphere; right hemisphere–left hemisphere; right hemisphere–right hemisphere) and echo time as factors, and the *n* = 20 subjects’ FC increases as the multiple dependent variables. FC increases were averaged across all connections in the indicated quadrant of the connectivity matrix. Post hoc paired 2-sample *t*-tests (*n* = 20) on Fisher-transformed correlation coefficients were conducted to identify the hemispheres and echo times exhibiting significant differences in FC enhancement.

### MR Thermometry Preprocessing

Brain temperature recordings followed a similar preprocessing procedure as the BOLD signals. The AfNI function *align_epi_anat.py* was employed to perform the combined operations of slice time correction, motion correction, and registration of temperature volumes to the anatomical images. We used the *lpa* cost function and registered the magnitude of the PRF to define the transformation, which was then applied to the phase data. Phase difference images were smoothed with a FWHM of 8 mm using *3dBlurInMask*. We employed the PRF equation to convert the phase differences to temperature changes:

$$ \Delta T=\frac{\Delta \varphi}{{a\gamma B}_o TE}, $$

where ∆*T* (units of °C) is the temperature change from baseline, ∆φ (units of radians) is the phase difference from baseline, *a* = *−*0*.*01 *×* 10^*−*6^ is the PRF constant modeling the sensitivity of the resonance frequency to temperature changes, *B*_0_ = 3 T, TE = 0*.*017 s, and γ = 2π *×* 42*.*58 *×* 10^6^ rad/s. We then regressed out the time series of the *B_o_* reference (located in the cerebellum) as well as the time series of the motion parameters and their derivatives. Voxels whose mean signal power was >4 standard deviations (SD) above the mean of all voxels were censored.

### MR Thermometry Analysis

In order to ensure the accuracy of the MR thermometry sequence and subsequent data analysis, we conducted recordings with an agar phantom that was heated up with the laser at a high intensity. Ground-truth temperature in the phantom was simultaneously measured with an infrared temperature sensor and used to validate the MR-derived temperature. To test for the presence of heating during the human tPBM experiments, we averaged the temperature change (from baseline) across all gray matter voxels in the illuminated region (including the white matter produced negligible changes, data not shown). This was computed separately for all time points and both active and sham conditions. We then performed paired 2-tailed Wilcoxon signed-rank tests with *n* = 20 to detect time points during which there was a significant difference in temperature change between active and sham stimulation. Correction for multiple comparisons was implemented by controlling the FDR at 0*.*05.

## Results

**Figure 1:**
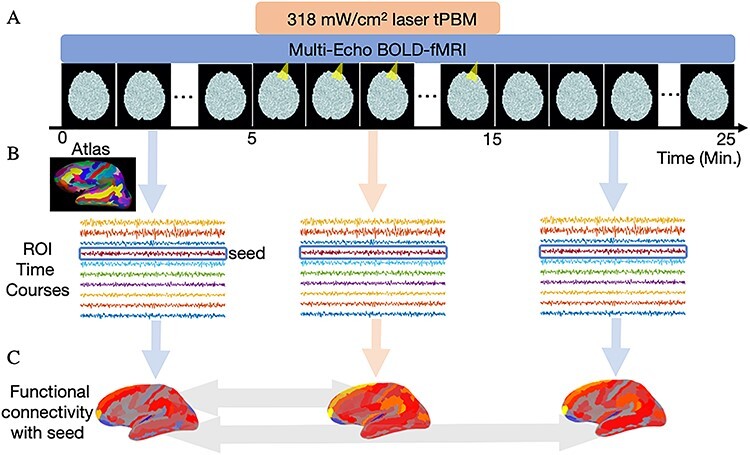
Experimental design. (*A*) To identify the effect of tPBM on hemodynamic activity in the human brain, we recorded the BOLD-fMRI signal from *n* = 20 healthy participants before, during, and after illumination. tPBM was applied with an 808-nm laser at an intensity of 318 mW/cm^2^ beginning 5 min after the start of the 25-min analysis window. The duration of tPBM was 10 min. (*B*) BOLD signals were converted into ROI time series corresponding to 151 cortical regions from the Destrieux atlas (Destrieux et al. 2010) and the illuminated region. (*C*) FC was computed as the Pearson correlation between the time courses of a “seed” region and connecting ROIs. We measured the difference in FC between the illumination and preillumination periods, and also between the postillumination and preillumination periods (indicated with light gray arrows). Permutation tests were conducted to test for significant changes in FC, with multiple comparisons corrected by controlling the FDR at 0.05.

To investigate the effect of tPBM on hemodynamic activity and temperature in the human brain, we conducted experiments combining laser stimulation with fMRI in healthy human participants at rest. tPBM was applied to the right frontal pole (scalp location Fp2) of *n* = 20 subjects with a monochromatic 808-nm laser at an intensity of 318 mW/cm^2^ and a 10-min duration. A multi-echo (13, 34, and 55 ms) BOLD-fMRI sequence was employed to capture hemodynamic changes due to tPBM, with a 25-min analysis window consisting of the 5 min leading up to illumination, the 10-min stimulation period, and the 10 min immediately after illumination (Fig. 1).The inclusion of multiple echoes allowed us to probe early BOLD changes that may be driven by modulations of CBF. To determine whether tPBM produced significant heating of the brain, we then employed MR thermometry to measure brain temperature in a separate cohort of *n* = 20 participants receiving tPBM with the same dose as the BOLD-fMRI study. In both studies, no subjects reported perceiving sensations from illumination, either thermal or otherwise, following the experiments.

An individualized ROI was defined for each subject based on the precise scalp site of illumination and a simple model of transcranial light propagation (see Materials and Methods). The cylindrical ROI extended approximately 2 cm radially and 4 cm axially, designed to contain at least 99% of the total light absorption. In what follows, we refer to this ROI as the illuminated region.

### Near-Infrared Light Increases FC with the Illuminated Region

Temporal correlations between the hemodynamic activity of different brain regions have been widely observed in the resting states and comprise the basis of FC (Fox and Raichle 2007). We suspected that the near-infrared stimulation would increase the FC between the illuminated region and its connected neighbors. To test this, we measured FC before, during, and after illumination. Connectivity analysis was carried out on a set of 151 ROIs formed as the union of the Destrieux brain atlas (Destrieux et al. 2010) and the illuminated region.

**Figure 2:**
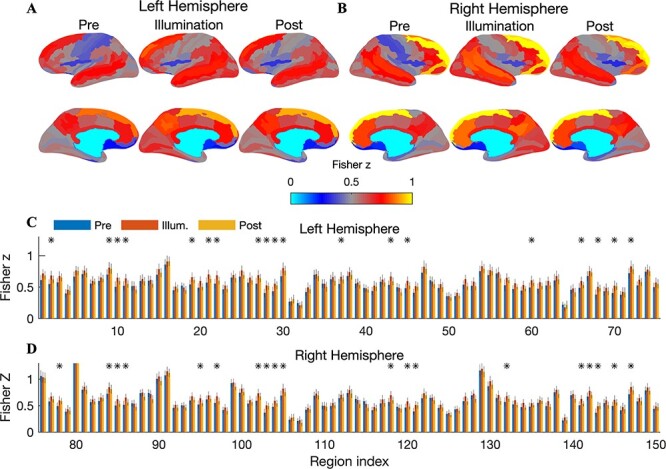
Increased FC with the illuminated region during tPBM (echo 3). (*A*) Cortical surfaces display the group-averaged FC (Fisher transformed Pearson correlations) between the illuminated region and all left hemispheric ROIs, shown separately for the preillumination, illumination, and postillumination periods. During tPBM, increased connectivity with ROIs in the frontal and parietal lobes was readily apparent. The magnitude of the increase was reduced following illumination. (*B*) Same as *A* but showing FC with the right hemisphere. Increased connectivity with regions in the frontal, temporal, and parietal lobes was observed. (*C*) Bar graphs show Fisher-transformed correlation coefficients between the illuminated region and each cortical region in the left hemisphere. Error bars depict the SEM across *n* = 20 subjects. Connections that exhibited statistically significant increases during illumination are denoted with a black asterisk (permutation test, corrected for multiple comparisons by controlling the FDR at 0.05). In total, 19 of the 75 connections (25%) showed a significant increase. (*D*) Same as *C* but now for the right hemisphere. In total, 19 of the 75 connections exhibited a significant increase during illumination. ROI labels for all significantly enhanced connections are provided in Supplementary Table S2.

We first compared FC between the illuminated region and all other ROIs across the 3 temporal segments of the experiment. During illumination, a robust increase in connectivity was observed at all echo times (results for echo 3 shown in Fig. 2*A*,*B*). FC was visibly increased in the frontal and parietal cortices of both hemispheres. Similar increases in FC were also found at echoes 1 and 2 (Supplementary Figs S2 and S3). Of the 150 connections that the illuminated region made with other regions, 38 (19 significant connections with the left hemisphere, 19 with the right) exhibited a statistically significant increase during illumination relative to the prestimulation period (permutation test with phase-randomized surrogate data records modeling static connectivity, corrected for multiple comparisons by controlling the FDR at 0.05; shown for echo 3 in Fig. 2*C*,*D*). The labels corresponding to the ROI indices in Figure 2*C*,*D* are listed in Table 1 of Destrieux et al (2010). At echoes 1 and 2, the number of significantly enhanced connections was 18 and 16, respectively. Averaged across all connections and subjects, the percent increases in FC were 6.35 *±* 5.00%, 4.30 *±* 4.97%, and 6.66 *±* 5.23% at echoes 1, 2, and 3, respectively (means *±* standard error of means [SEMs] across *n* = 20 subjects). At echo 3, the largest measured increase of 15% was with the left posterior cingulate. Other significantly increased connections were with the right superior parietal gyrus, the right postcentral gyrus, the right subparietal sulcus, and the right cuneus. Only a few connections exhibited significant increases in the postillumination period, all at echo 1: the dorsal posterior cingulate of both left and right hemispheres and the subparietal sulcus in the left hemisphere. The connecting ROIs that exhibited significant increases during and after illumination are listed in Supplementary Table S2 for all echoes.

**Figure 3:**
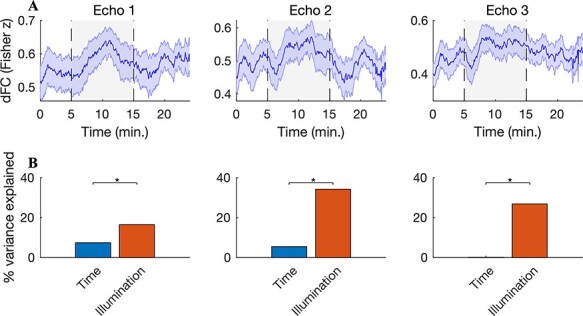
Increased dFC during illumination. (*A*) A 2-min sliding window was employed to measure dFC with the illuminated region (averaged across all 150 connecting ROIs and *n* = 20 subjects; shaded error bars depict the SEM across subjects). A sharp increase in dFC was observed shortly after illumination onset for all echo times. The dFC increase was somewhat reduced towards the latter portion of the illumination period—note that the sliding window extends into the postillumination period in the final 2 min of illumination. (*B*) To test for a possible effect of time on the observed dFC changes, we separately regressed dFC onto (i) time and (ii) a boxcar regressor modeling the time course of illumination. At all echo times, the illumination time course explained a significantly larger proportion of dFC variance: 17%/34%/27% compared with 7%/5%/*<*1% for echoes 1, 2, and 3, respectively (*P <* 1 × 10^*−*13^, Fisher *r*-to-*z* test comparing Pearson correlation coefficients between the regressor and thedFC).

To probe the dynamics of the increased FC, we measured the time course of connectivity by employing a sliding window of length 2 min and a 1-TR shift between successive windows. This measure is often termed “dFC” (Hutchison et al. 2013). To minimize the number of statistical comparisons, here we considered total connectivity, which we define as the mean correlation between the illuminated region and its connections (i.e., mean across 150 connecting ROIs). We observed a sharp increase in dFC approximately 1 min after illumination onset for all echo times (Fig. 3*A*). The increased dFC was somewhat reduced toward the latter part of the 10-min illumination period, perhaps partly due to the sliding window computation that is required to measure dFC (in the last 2 min of illumination, this window extended into the postillumination period).

In order to test for a possible effect of time on the observed changes in dFC (i.e., spontaneously increasing connectivity that is not attributable to the stimulation), we measured the proportion of dFC variance explained by a simple regression modeling increasing time. The passage of time explained 7%, 5%, and *<*1% of the dFC variance in echoes 1, 2, and 3, respectively. To contrast this with the effect of the stimulation, we performed a second regression with a boxcar regressor modeling the time course of illumination: off for 5 min, on for 10 min, then off again for 10 min. This model explained 17%, 34%, and 27% of the dFC variability at echoes 1, 2, and 3, respectively. The proportion of variance explained by the illumination time course was significantly larger than that explained by time at all echoes (Fisher *r*-to-*z* test comparing the Pearson correlation coefficient between the regressor and the dFC; *z >* 7*.*43, *P <* 1 *×* 10^*−*13^).

### Brain-Wide Increases in FC

**Figure 4:**
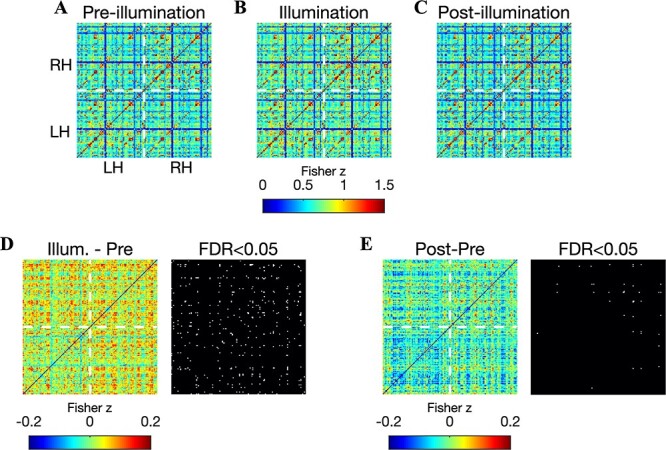
Brain-wide increases in FC during illumination (echo 3). Images show the correlation matrix between all pairs of 151 ROI time courses before (*A*), during (*B*), and after (*C*) illumination. The lower left and upper right quadrants indicate connections within the left and right hemispheres, respectively. The upper left and lower right quadrants indicate interhemispheric connectivity. Stimulation was delivered to the right frontal pole. (*D*) The difference between correlation matrices measured during and before illumination: a broad increase of up to 0.2 was readily observed, with a visible dampening of the increase in the lower left quadrant—left hemispheric connections were less affected. Binary image (right) indicates the connections that exhibited a significant increase (in white) during illumination (permutation test, corrected for 11 325 comparisons using the FDR at 0.05). A total of 283 significant connections were detected. (*E*) Same as (*D*) but now between the postillumination and preillumination periods. A total of 32 connections exhibited a statistically significant increase after illumination, with a majority of these located within the right hemisphere.

To probe the potential effects of tPBM on connectivity between brain areas outside of the illuminated region, we measured correlation matrices encompassing all connections among the 151 ROIs for the three segments of the experiment (shown for echo 3 in Fig. 4*A*,*C*). For the cortical ROI set employed in our analysis, we observed that the group-averaged FC matrices were strictly positive. However, anticorrelations were observed in the FC matrices of individual subjects (data not shown). The illumination period was marked by a pronounced connectivity increase at numerous segments of the correlation matrix, in particular the 3 quadrants corresponding to connections with ROIs in the stimulated right hemisphere (Fig. 4*D*). Notice the relative absence of increases in the lower-left quadrant, which corresponds to connections within the left hemisphere. In all, 283 connections (2.5%) exhibited statistically significant increases during the illumination period at echo 3; 116 and 259 were found at echoes 1 and 2, respectively (Supplementary Figs S4 and S5). The increase was largely dampened following illumination (Fig. 4*E*). Nevertheless, we did detect a small number of connections (i.e., 32 at echo 3) exhibiting a significant increase after illumination (Fig. 4*E*). There were 44 and 7 significant postillumination connections detected at echoes 1 and 2, respectively.

**Figure 5:**
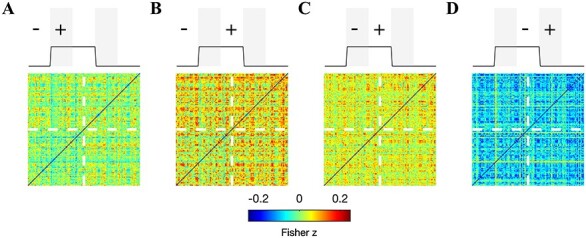
Accumulation of brain-wide FC throughout the illumination period. Images depict FC difference matrices between: (*A*) the first 5 min of illumination and the 5-min preillumination window, (*B*) the second 5 min of illumination and the 5-min preillumination window, (*C*) the second 5 min of illumination and the first 5 min of illumination, and (*D*) the first 5 min after illumination and the second 5 min of illumination. All matrices are shown for echo 3. Notice that, at many connections, FC is continuing to increase during the latter half of illumination.

To gain insight into the dynamics of the global FC increases, we also measured brain-wide FC in 5-min windows. We then computed difference matrices to identify early and late changes in FC (shown for echo 3 in Fig. 5*A*–*D*). Brain-wide increases were accumulating during the 10-min illumination period, with large portions of the FC matrix continuing to increase in the latter half of the illumination period (Fig. 5*C*). The reduction in FC following illumination was observed in the first 5-min poststimulation (Fig. 5*D*).

**Figure 6:**
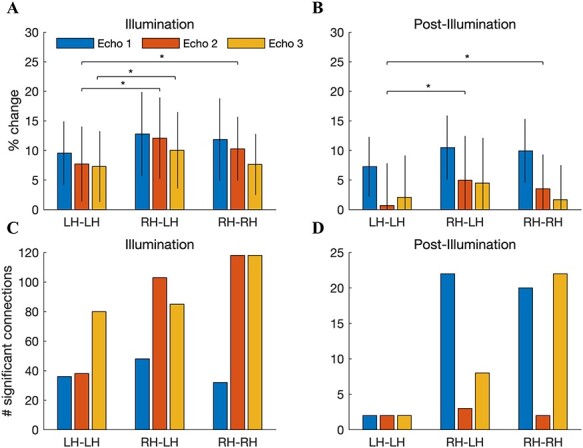
Increased FC is more pronounced in the stimulated hemisphere. (*A*) The percent increase in FC, depicted separately for connections within the left hemisphere, between the left and right hemispheres, and within the right hemisphere. The percent change was averaged across all connections in the specified region. Error bars denote the SEM across *n* = 20 subjects. A repeated-measures ANOVA with hemisphere and echo time as factors and the acute FC increase as the dependent variables revealed main effects of both echo time (*F*(19) = 14*.*4, *P* = 2*.*9 *×* 10^*−*20^) and hemisphere (*F*(19) = 2*.*16, *P* = 0*.*0079). Post hoc paired *t*-tests showed that the hemispheric preference was found at echoes 2 (RH–LH vs. LH–LH; RH–RH vs. LH–LH) and 3 (RH–LH vs. LH–LH). (*B*) After illumination, main effects of echo time (*F*(19) = 12*.*55, *P* = 2*.*6 × 10^*−*18^) and hemisphere (*F*(19) = 3*.*71, *P* = 1*.*09 × 10^*−*5^) were also observed. Post hoc *t*-tests showed that the effect was focused at echo 2. (*C*) The number of significantly enhanced connections during illumination also exhibited a preference for the stimulated hemisphere. (*D*) This trend was sustained after illumination.

We suspected that the stimulation would preferentially modulate connectivity with regions in the stimulated (right) hemisphere. To test this, we computed the FC increase separately for connections within the left hemisphere (LH–LH), between the right and left hemispheres (RH–LH), and within the right hemisphere (RH–RH). We then conducted a 2-way, repeated-measures ANOVA with hemisphere and echo time as factors, and the percent change in FC during illumination as the dependent variable. We found a significant main effect of echo time (*F*(19) = 14*.*4, *P* = 2*.*9 *×* 10^*−*20^) and, importantly, hemisphere (*F*(19) = 2*.*16, *P* = 0*.*0079). We did not observe an interaction between echo time and hemisphere. Post hoc paired *t*-tests showed that the FC increase was stronger in RH–LH compared with LH–LH at echoes 2 and 3 (Fig. 6*A*; *P* = 0*.*0012 and *P* = 0*.*037, respectively, paired 2-sample *t*-test, *n* = 20) and stronger in RH–RH compared with LH–LH at echo 2 (*P* = 0*.*032).

The preference for the stimulated hemisphere was sustained after illumination. We again observed main effects of echo time (*F*(19) = 12*.*55, *P* = 2*.*6 *×* 10^*−*18^) and hemisphere (*F*(19) = 3*.*71, *P* = 1*.*09 *×* 10^*−*5^). Post hoc *t*-tests showed that the effect was focused at echo 2 (RH–LH vs. LH–LH: *P* = 0*.*0031; RH–RH vs. LH–LH: *P* = 0*.*020; Fig. 6*B*).

We also found that the number of significantly enhanced connections was higher for connections involving the right hemisphere; for example, during illumination at echo 2, 32 significant connections were found within the left hemisphere, whereas 118 were found in both RH–LH and RH–RH connections (Fig. 6*C*). Although the number of significant connections dropped dramatically following illumination, this trend was still evident (Fig. 6*D*).

### No Evidence for Brain Temperature Increase with MR Thermometry

**Figure 7:**
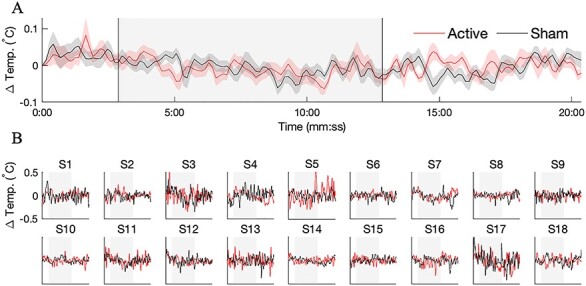
No evidence for brain heating with MR thermometry. A separate cohort of *n* = 20 subjects was recruited for a study aimed at resolving temperature changes during tPBM with the same dose as the BOLD study. (*A*) Group-averaged temperature in the illuminated region, shown separately for active (red) and sham (black) stimulation. Error bars depict the SEM across *n* = 20 subjects. We failed to detect any time points during which the temperature in the illuminated region was significantly different during active versus sham stimulation (paired Wilcoxon signed-rank test, *n* = 20, corrected for multiple comparisons by controlling the FDR at 0.05) (*B*) Temperature time courses for individual subjects. In all, 2 of the 20 subjects were excluded due to excessive recording artifacts. Despite the presence of drifts and spurious fluctuations, the temperature time series of active and sham stimulation largely overlapped.

We conducted a subsequent study to test for the presence of brain temperature increases during tPBM. A separate cohort of *n* = 20 healthy subjects participated, with tPBM applied at the same dose as in the BOLD-fMRI study. Subjects performed 2 sessions—active and sham stimulation—in succession, with the order of active and sham randomized and balanced across subjects. We employed an MR thermometry sequence that exploits the temperature sensitivity of the resonance frequency (Rieke and Butts Pauly 2008; Od́een et al. 2014). A 2-min baseline period preceded the 10-min illumination which was followed by an additional 8 min of recording. For both sham and active stimulation, we measured the temperature in the illuminated region at a temporal resolution of one measurement every 8.2 s (Fig. 7). We did not detect significant temperature differences between active and sham stimulation at any time point before, during, or after illumination (Wilcoxon signed-rank test, *n* = 20, corrected for multiple comparisons by controlling the FDR at 0.05). The maximum absolute temperature difference between active and sham stimulation was *−*0*.*14^°^C (i.e., a relative temperature decrease with active stimulation) that occurred 122 s after the onset of illumination (Fig. 7*A*). The time series of temperature in the illuminated region of individual subjects showed a high amount of overlap between active and sham stimulation (Fig. 7*B*). We also tested for potential differences in the variability of brain temperature. The measured temperature fluctuations were very similar for active and sham stimulation (active: SD = 0.14*°*C during stimulation; sham: SD = 0.13*°*C; SDs averaged across *n* = 20 subjects).

## Discussion

We employed BOLD-fMRI to measure hemodynamic activity in the brains of healthy human participants as they received transcranial stimulation with a near-infrared laser directed at the right frontal pole for 10 min. We found a robust increase in FC with the illuminated region, indicating that hemodynamic activity in the stimulated area became more synchronized with other brain regions during illumination. The increase emerged approximately 1 min after illumination onset. We also found increased connectivity between regions outside of the directly stimulated area, with a more pronounced effect in connections involving the stimulated hemisphere. These main findings were observed at all 3 echo times, with only minor variations in number and effect size. We then measured brain temperature during laser stimulation with MR thermometry, finding no significant temperature differences between active and sham stimulation.

A limitation of our study is the difficulty in inferring the precise origin of the increased FC during illumination. The BOLD signal is a complex mixture of effects from changing CBF, CMRO_2_, and the arterial concentration of oxygen (Buxton 2013). Therefore, it is challenging with the present data to disambiguate a direct vascular effect (CBF) from an indirect effect due to neural activation (CMRO_2_). Given our finding of increased FC at an early echo (13 ms), it is likely that increased CBF was involved in the observed BOLD changes—the BOLD contrast due to oxygenation is very weak at this short echo time. An inflow of fully relaxed hemoglobin into the region would increase the initial value of the T2^*^ signal, producing a signal increase at short echo times such as what was observed here (Posse et al. 1999; Kundu et al. 2012). On the other hand, several aspects of our findings indicate that the effect of the stimulation was strongest at the later echoes. For example, the number of significantly enhanced connections with the illuminated region was highest for echo 3 (Fig. 2), and the laser time course explained more dFC variance at echoes 2 and 3 than at early echo 1 (Fig. 3). These findings suggest that cerebral oxygenation was also modulated, as the change in the BOLD contrast due to CMRO_2_ increases linearly with echo time. Therefore, it seems likely that both CBF and CMRO_2_ were modulated during tPBM.

One aspect of the present findings that points to neural activity changes during tPBM is the pattern of brain regions that was modulated. Many of the connections enhanced during illumination (e.g., posterior cingulate cortex, precuneus; see Supplementary Table S2) belong to the “default-mode network,” a set of regions preferentially activated in the absence of a task (Van Den Heuvel and Pol 2010; Sheline et al. 2009). By itself, a local vascular effect at the illuminated region would seem unlikely to manifest in hemodynamic correlations across such a distributed set of brain areas. Nevertheless, to tease apart vascular and metabolic changes during tPBM, additional studies with alternative MR techniques such as arterial spin labeling, which measures CBF (El Khoury et al. 2019) or magnetic resonance spectroscopy, which measures metabolism, are needed.

Previous reports of tPBM found increases in oxygenated hemoglobin with NIRS (Tian et al. 2016) and increased power of alpha band (8–12 Hz) EEG oscillations. The large increase in FC at the latest echo time found here suggests that oxygenation was involved, consistent with Tian et al. (2016). Moreover, associations between electrophysiological oscillations and hemodynamic activity of resting-state networks have been previously identified (Mantini et al. 2007). The present findings complement the recent reports of increased connectivity following LED-based tPBM (El Khoury et al. 2019; Chao 2019; Naeser et al. 2019). Here we employed a monochromatic laser to produce robust acute increases in connectivity, which likely mediate the previously reported outlasting effects.

The findings of the MR thermometry study suggest that brain temperature does not significantly change during tPBM. The fluctuations that were observed here were within the range observed in the brains of patients following head injury, where direct measurements are feasible (Soukup et al. 2002; Wang et al. 2014). Moreover, the variability in temperature measured during tPBM (0.13–0.14*°*C) is similar to the temperature changes estimated from a model linking brain temperature with the BOLD signal (0.2*°*C) (Yablonskiy et al. 2000). It therefore seems unlikely that the observed FC increases were mediated by a thermal effect. The absence of brain heating supports CCO as the relevant chromophore in tPBM—the most probable alternative, water, is abundant in the brain and absorption of light energy by water molecules may have led to measurable temperature changes. The PRF technique employed here to measure temperature is known to be sensitive to drift of the static magnetic field and subject motion (Rieke and Butts Pauly 2008). We employed signal processing measures to mitigate these influences. However, it is possible that brief, acute heating followed by immediate increased CBF occurred and was not detectable within the temporal and temperature resolution of our measurements. In the future, we encourage the employment of newer sequences for MR thermometry that can achieve finer precision in temperature estimation (Od́een and Parker 2019). These studies will be invaluable to either conclusively rule out heating, or to identify small changes that eluded the methodology employedhere.

## Notes

The authors would like to acknowledge Henrik Odeen and Siemens for the collaborative Work-in-progress package (WIP 1118-VE11C, Version 1.0, November 2017) research pulse sequence that was employed in the MR thermometry experiments. The authors would also like to thank Lazar Fleysher for technical support during the initial development of the experimental setup. We would also like to acknowledge the help of Christian Fong in subject recruitment and experimental assistance, as well as helpful preliminary discussions with Hanli Liu. The authors would like to acknowledge the Magnetic Resonance Imaging Facility of CUNY Advanced Science Research Center for instrument use and technical assistance. *Conflict of Interest*: None declared.

## Funding

This work was supported by the City University of New York (CUNY) Junior Faculty Research Award and the Translational Research Institute through NASA Cooperative Agreement NNX16AO69A. 

## Supplementary Material

supplement_tgaa004
