# Supplementary material for: Near-Infrared Light Increases Functional Connectivity with a Non-thermal Mechanism
Source: Cereb Cortex Commun. 2020 Mar 19;1(1):tgaa004. doi: 10.1093/texcom/tgaa004 (PMC8152883; doi:10.1093/texcom/tgaa004)
Supplement: supplement_tgaa004 [file supplement_tgaa004.pdf]

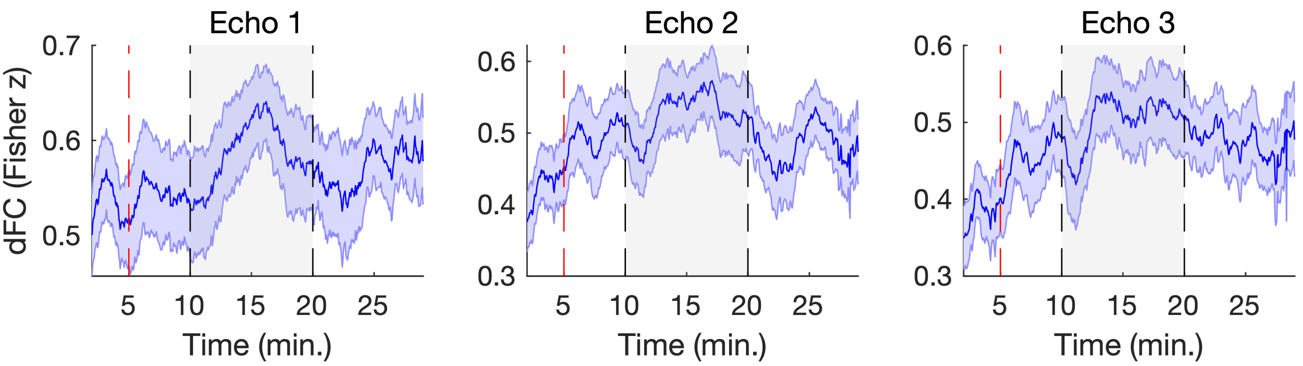

Figure S1: Exclusion of first 5 minutes of BOLD due to transient functional connectivity. The time course of total connectivity with the illuminated region for Echo 1 (left), Echo 2 (middle), and Echo 3 (right). Connectivity exhibited a transient drift during the first five minutes of the pre-illumination period before reaching a stable value at approximately five minutes (indicated with vertical dashed red line). Consequently, we excluded the first five minutes of the baseline period from further analysis.

**Layer**	**Refractive Index**	**Absorption Coefficient** (μ_a)	**Scattering Coefficient** (μ_s)	**Anisotropy Factor** (g)	**Thickness (cm)**
Scalp	1.41	0.2	31.25	0.52	0.6
Skull	1.56	0.07	130	0.9	0.65
CSF	1.33	0.02	0.1	0.9	0.2
Brain	1.4	0.084	90	0.9	8

Table S1: Physical and optical properties of the four-layer head model employed in the simulation of light propagation. Whenever possible, we have used *in vivo* optical properties. In particular, while MCML requires separate specification of μ_s and g, *in vivo* values for the reduced scattering coefficient $\mu_s' \equiv \mu_s(1-g)$ and g are more readily available. As such, we have inferred values of μ_s from reported values of μ_s' and g. All values of μ_a and μ_s are in units of cm^{-1}.

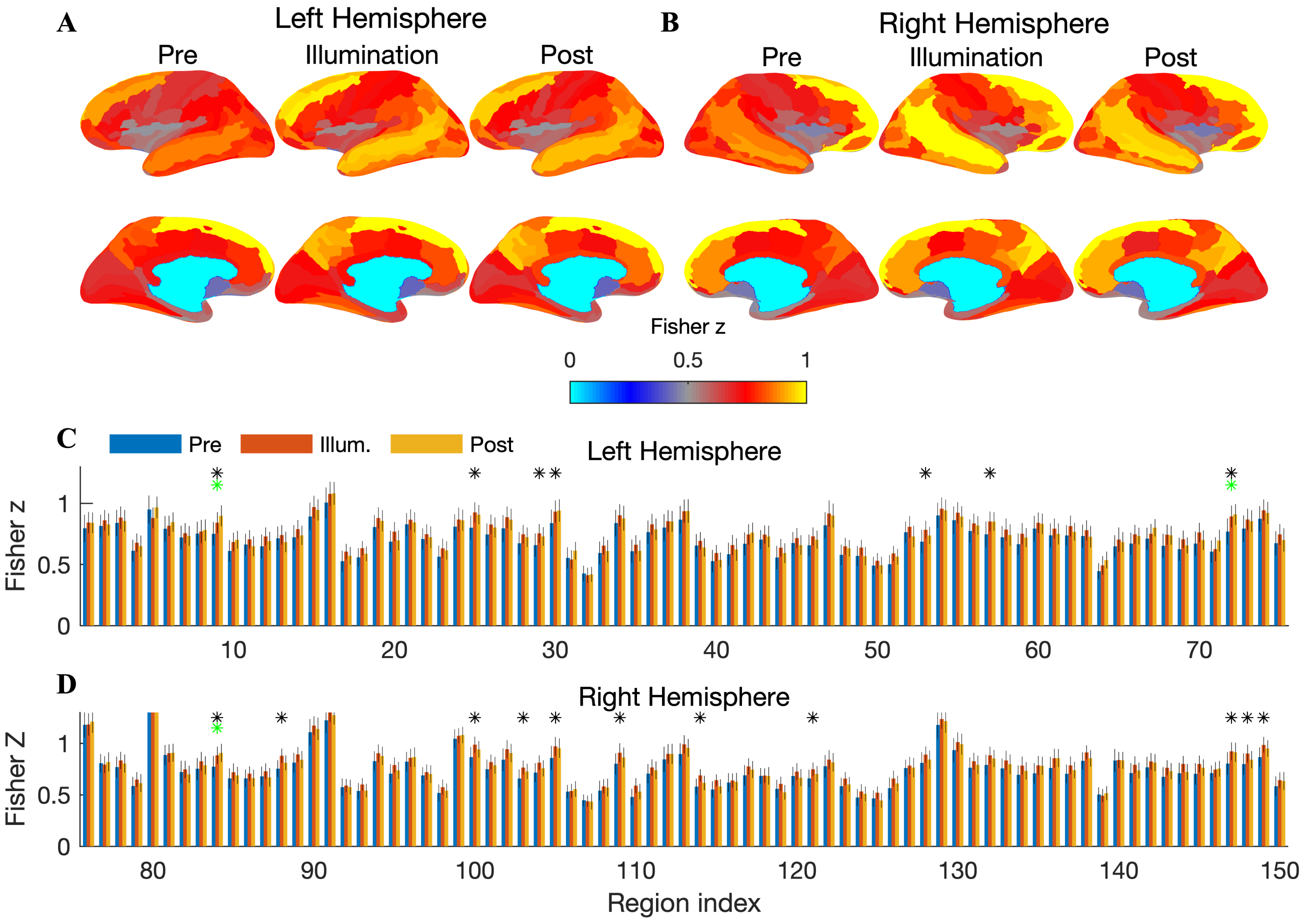

Figure S2: Increased functional connectivity with the illuminated region at Echo 1. (**A**) Cortical surfaces depict the Fisher transformed Pearson correlation between the illuminated region and all ROIs in the left hemisphere, shown separately for the pre-illumination, illumination, and post-illumination period. (**B**) Same as (A) but now showing connectivity between the illuminated region and ROIs in the right hemisphere. (**C**) Bar graphs depict the mean connectivity before, during, and after illumination for each ROI in the left hemisphere. Asterisks denote a significant increase during illumination. Green asterisks denote a significant post-illumination effect, which here was only observed with connections to three ROIs in the parietal cortices. (**D**) Same as (C) but now showing connectivity with the right hemisphere. Across both hemispheres, 18 ROIs exhibited a significant increase in connectivity with the illuminated region during illumination.

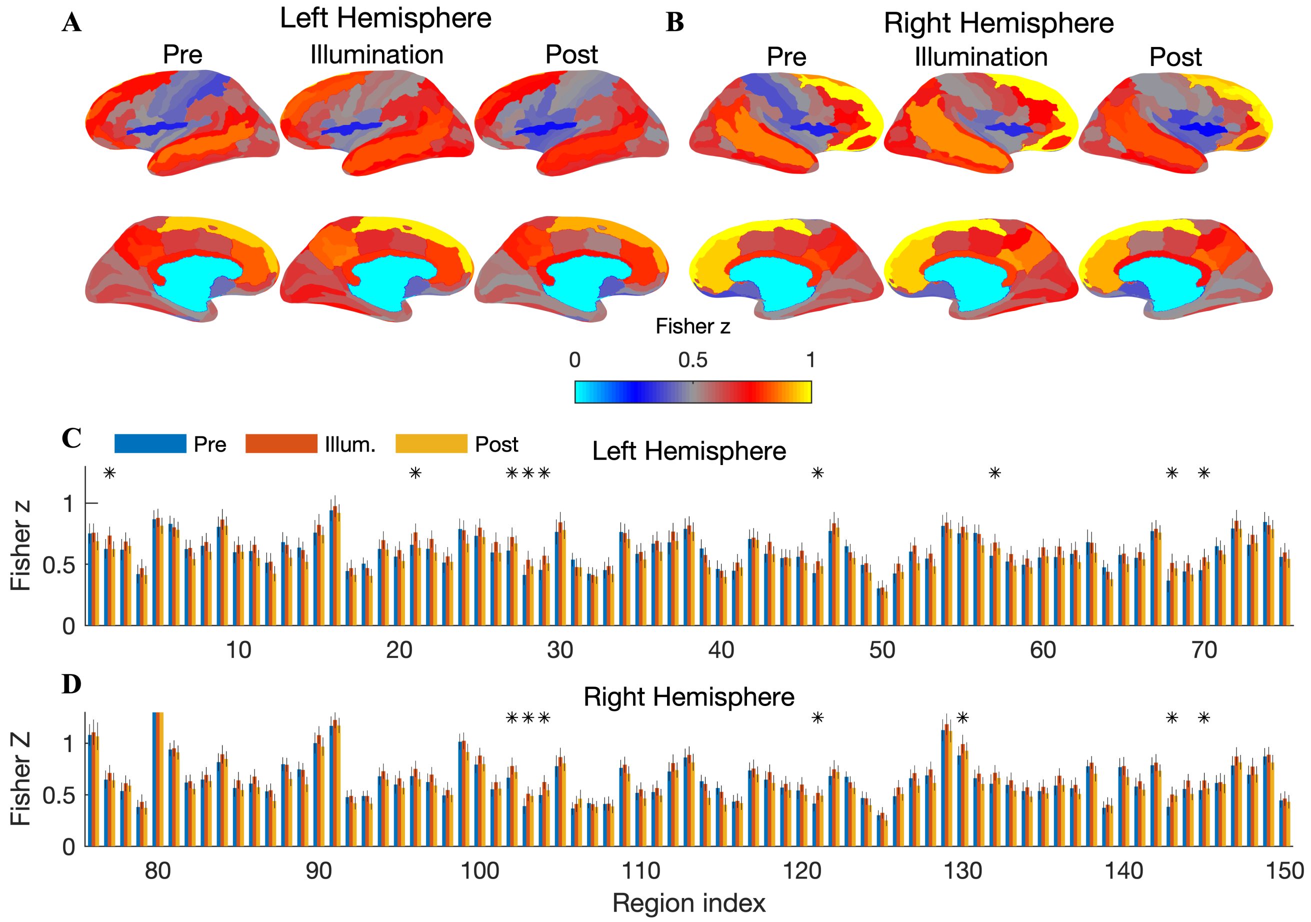

Figure S3: Increased functional connectivity with the illuminated region at Echo 2. Panels match those of Figure S2. A total of 16 ROIs exhibited a significant acute increase in connectivity with the illuminated region.

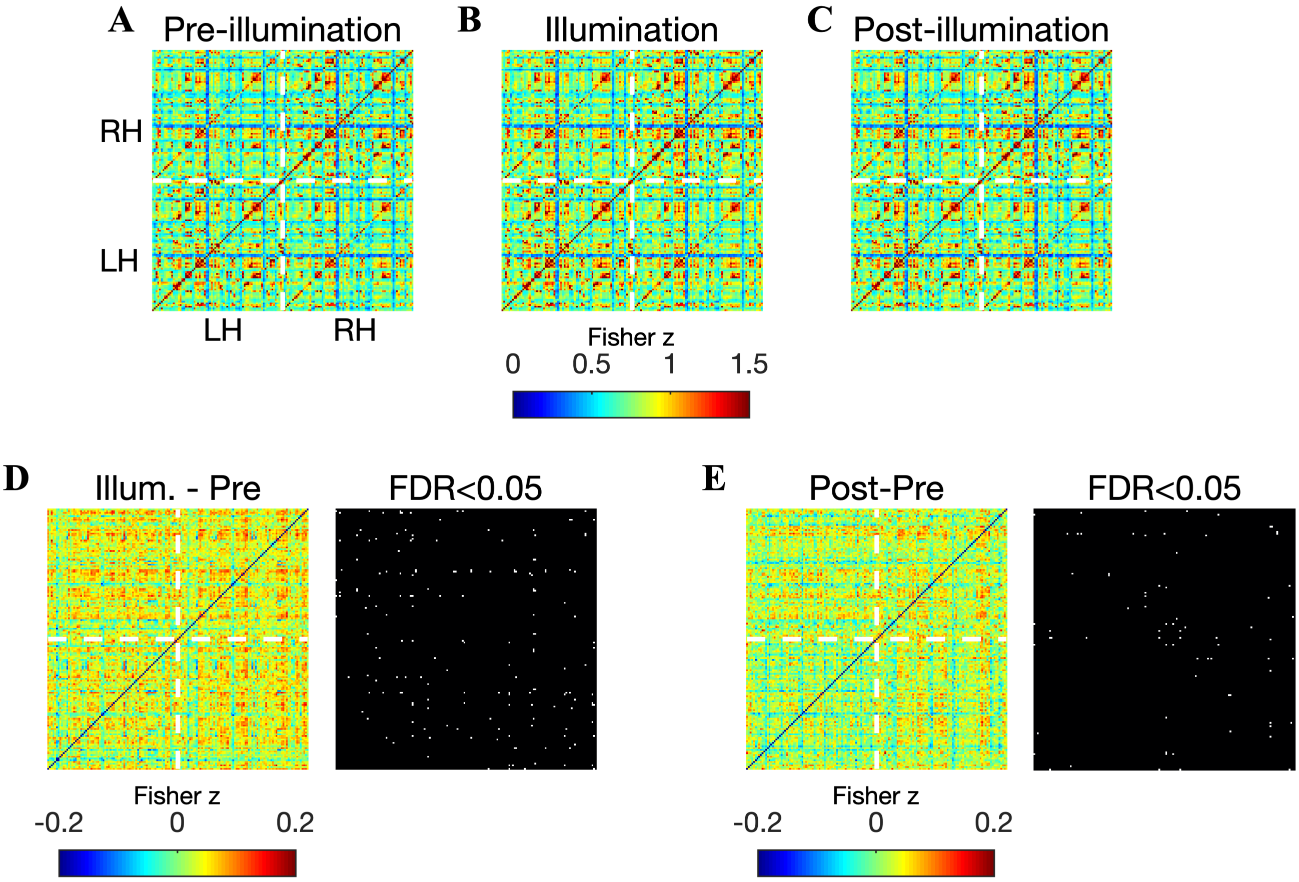

Figure S4: Increased functional connectivity with seeds outside of the illuminated region (Echo 1). (**A**) Matrix showing the Fisher transformed Pearson correlation between all pairs of 151 ROI time courses before illumination. (**B**) Same as (A) but now for the illumination period. (**C**) Correlation matrix for the period after illumination. (**D**) The difference in correlation matrices measured during and before illumination. 116 connections exhibited significant increases. (**E**) Same as (D) but now between the post- and pre-illumination periods. 44 significant connections were detected.

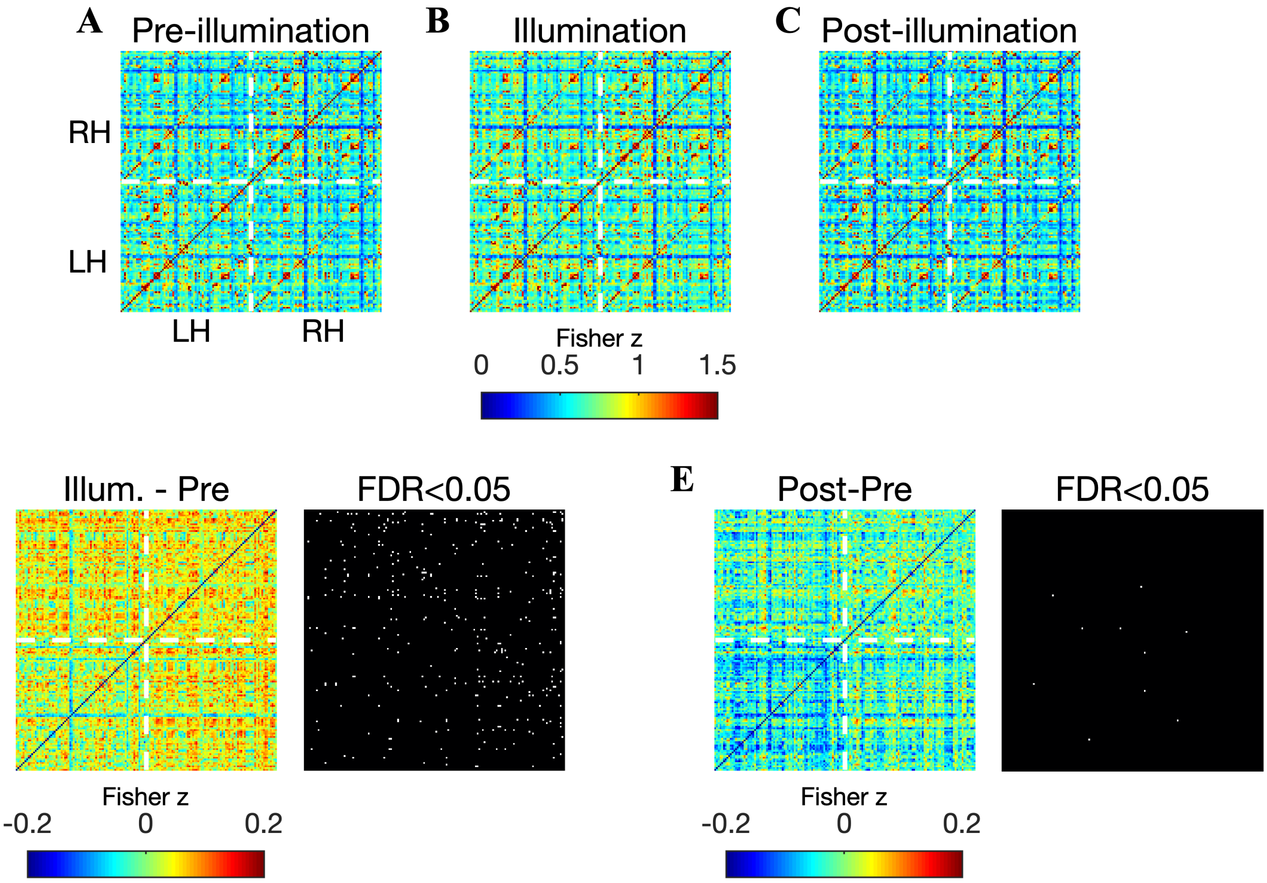

Figure S5: Increased functional connectivity with seeds outside of the illuminated region (Echo 2). Panels match those of Fig S4. 259 connections exhibited significant increases during illumination, while 7 showed increased connectivity following tPBM.

**Echo 1**	**Echo 2**	**Echo 3**
lh_G_cingul-Post-dorsal	lh_G_and_S_occipital_inf	lh_G_and_S_occipital_inf
lh_G_pariet_inf-Angular	lh_G_oc-temp_lat-fusifor	lh_G_cingul-Post-dorsal
lh_G_precentral	lh_G_parietal_sup	lh_G_cingul-Post-ventral
lh_G_precuneus	lh_G_postcentral	lh_G_cuneus
lh_S_front_inf	lh_G_precentral	lh_G_occipital_middle
lh_S_intrapariet_and_P_tran	lh_S_central	lh_G_oc-temp_lat-fusifor
lh_S_subparietal	lh_S_intrapariet_and_P_tran	lh_G_oc-temp_med-Lingual
rh_G_cingul-Post-dorsal	lh_S_postcentral	lh_G_parietal_sup
rh_G_front_inf-Orbital	lh_S_precentral-sup-part	lh_G_postcentral
rh_G_pariet_inf-Angular	rh_G_parietal_sup	lh_G_precentral
rh_G_postcentral	rh_G_postcentral	lh_G_precuneus
rh_G_precuneus	rh_G_precentral	lh_G_temporal_inf
rh_G_temp_sup-Lateral	rh_S_central	lh_Pole_occipital
rh_Lat_Fis-ant-Horizont	rh_S_front_sup	lh_S_calcarine
rh_S_central	rh_S_postcentral	lh_S_occipital_ant
rh_S_subparietal	rh_S_precentral-sup-part	lh_S_parieto_occipital
rh_S_temporal_inf		lh_S_postcentral
rh_S_temporal_sup		lh_S_precentral-sup-part
lh_G_cingul-Post-dorsal		lh_S_subparietal
lh_S_subparietal		rh_G_and_S_paracentral
rh_G_cingul-Post-dorsal		rh_G_cingul-Post-dorsal
		rh_G_cingul-Post-ventral
		rh_G_cuneus
		rh_G_occipital_sup
		rh_G_oc-temp_med-Lingual
		rh_G_parietal_sup
		rh_G_postcentral
		rh_G_precentral
		rh_G_precuneus
		rh_Pole_occipital
		rh_S_calcarine
		rh_S_central
		rh_S_intrapariet_and_P_tran
		rh_S_parieto_occipital
		rh_S_pericallosal
		rh_S_postcentral
		rh_S_precentral-sup-part
		rh_S_subparietal

Table S2: ROIs exhibiting significantly increased FC with the illuminated region. The labels correspond to short names of the cortical regions comprising the Destrieux atlas. For unabbreviated labels, please refer to Table 1 of Destrieux et al. (2010). Italicized entries denote a significant FC increase after illumination.
